# Left atrial function examination of healthy individuals with 2D speckle-tracking imaging

**DOI:** 10.3892/etm.2012.789

**Published:** 2012-11-01

**Authors:** JIZHU XIA, YULI GAO, QINGQING WANG, WENYAN MA

**Affiliations:** Department of Ultrasound, Affiliated Hospital of Sichuan Luzhou Medical College, Luzhou, Sichuan 646000, P.R. China

**Keywords:** echocardiograph, two-dimensional speckle tracking imaging, speckle tracking imaging, left atrial function

## Abstract

The aim of the present study was to determine the left atrial (LA) function of healthy individuals of various ages by examining their LA strain and time-to-peak (TP) using 2D speckle-tracking imaging (2DSTI). In addition, the study investigated the potential value of STI in clinical applications. The 142 volunteers were divided into three age groups, namely, young adult, middle-aged and elderly. Images were obtained from examining the volunteers via echocardiography, 2D apical four-chamber, apical two-chamber and apical left ventricular longitudinal views. Following the examinations, an STI technique was applied to acquire the strain curves of the LA wall, which determined the strains and TPs. LA strains of the three age groups showed that the strain of the inferior segment was higher compared with that of the central segment, whereas the central segment was higher than that of the superior segment. The inferior segment of the strain of the elderly group decreased as age increased compared with the young adult and middle-aged groups. The central segment of the elderly group was lower than the young adult and middle-aged groups. As age increased, the TP of the inferior segment showed longer duration in the elderly group compared with the other two groups. The elderly and the middle-aged groups showed a longer central TP compared with the young adult group. No statistical significance was observed between the elderly and middle-aged groups. STI demonstrated objective and accurate examination results of the LA function, which provide a novel approach for the early monitoring of potential subclinical diseases in LA function.

## Introduction

Left atrial (LA) function plays a critical role in the cardiac cycle, particularly in the left ventricular (LV) filling and stroke volume during LV diastolic dysfunction, including the ventricular systole right ventricle (RV) function (reservoir), early ventricular diastolic conduit function (conduit) and ventricular diastolic ejection fraction (EF) function (booster pump) (1). Thus, the importance of LA is clear. Previously, the methods used to assess LA included motion-mode ultrasonography of LV size, ultrasonography area-length measurement of LV volume, spectral Doppler echocardiography of pressure of LA, LAEF pressure left atria (PLA), LAEF, pulmonary vein flow parameters (PVFP), acoustic quantification technique (AQ) of LA volume, quantitative tissue velocity imaging, 3D ultrasound and strain rate imaging methods. These methods have limitations in showing a complete view of the LA function, which imposes great challenge in performing LA function measurement.

Two-dimensional speckle tracking imaging (2DSTI) provides the velocity, strain and strain rate of the myocardium with 2D echocardiography.

Studies exist with regard to the STI of LA function (2,3), but LA studies on different age groups are lacking. The current study concentrates on 2D LA strain and time-to-peak (TP) measurements of healthy subjects of various age groups. The present study analyzes the regular correlation of 2D LA strain and TP of healthy adults of different ages and the potential of STI in clinical applications.

## Subjects and methods

### Subjects

A total of 142 healthy volunteers (80 male and 62 female) were selected between July 2010 and October 2011. The 142 volunteers were divided into age groups. The volunteers aged between 18 and 45 years accounted for 52 cases and belonged to the young adult group. The middle-aged group consisted of 49 cases, aged between 46 and 64 years. The elderly group consisted of 41 cases, whose ages were ≥65 years and averaged 70.40±4.76.

### Conventional echocardiography

During examination, the volunteers were asked to lie down in the left lateral position and to breathe normally. A synchronous Electrocardiogram (ECG) was connected to each volunteer to assess the left ventricular end systole (LVES) and left ventricular end diastole (LVED) from the parasternal longitudinal LV view. Biplane Simpson’s method was used to measure LVEF and apical four-chamber (Ap4C) for mitral forward flow and ratio of peak early to peak atrial (E/A ratio).

### 2DSTI tracking

Three sets of 2D echocardiography images of Ap4C, apical two-chamber (Ap2C) and LV longitudinal view were obtained and stored for offline analysis. During the QLAB software (version 8.1; Philips Ultasound, Bothell, WA, USA) analysis, samples were obtained at anterior, posterior, lateral and inferior walls of LA, atrial septa superior, central and inferior segments [where the superior segment was taken close to pulmonary vein (PV), the inferior segment was extracted at the mitral valve annulus, and the central was taken in between], and the myocardium inferior membrane. Samples were then measured to obtain the peak strain (PS) and TP of each segment as well as the average peak value (4).

### Statistical analysis

SPSS 13.0 (SPSS, Chicago, IL, USA) was used to analyze the data (expressed as mean ± standard deviation). One-way ANOVA was used to obtain P-values. P<0.05 was considered to indicate a statistically significant result.

## Results

### Volunteers

Variances of height, weight, cardiac rate, LVED and LVES diameters and LVEF of the healthy volunteers from the three groups did not show statistical significance (P_ave_>0.05). Compared with the young adult group, the mitral E/A of the middle-aged and elderly groups decreased. By contrast, Epeak deceleration increased, demonstrating a significant variance (P<0.05; Table I).

### LA strain and TP changes in healthy volunteer segments

**Figure 1:**
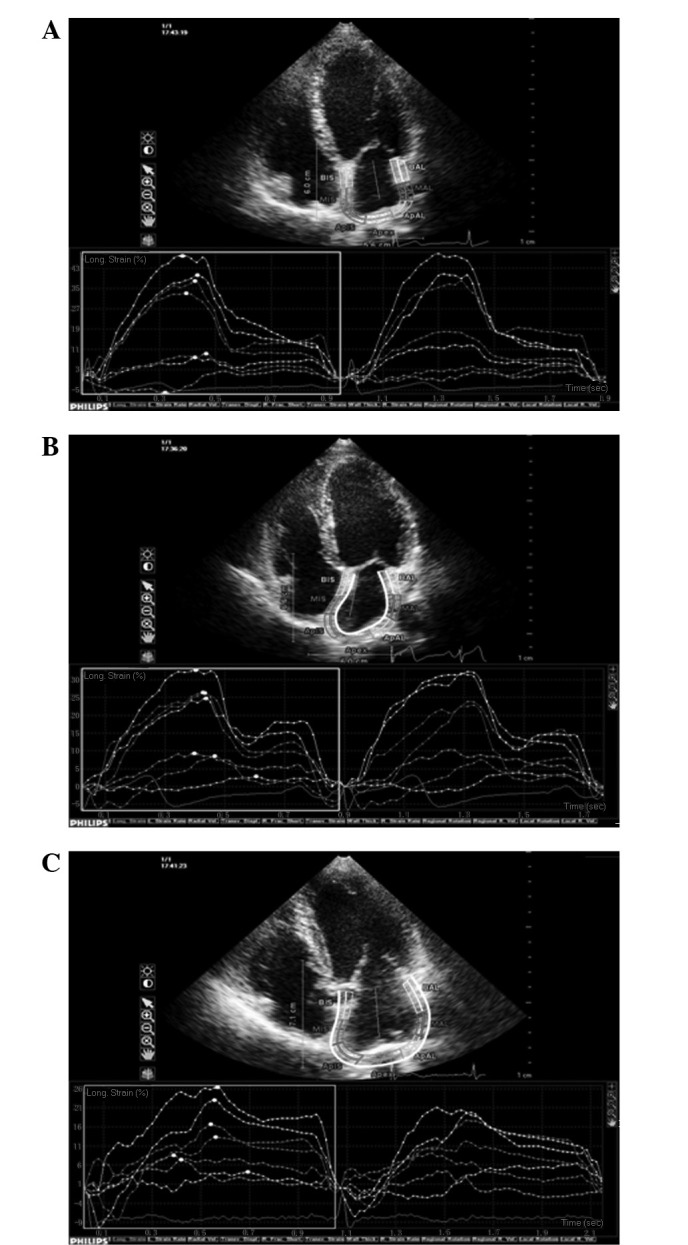
Strain course of left atrial longitudinal strain by speckle tracking. (A) The strain course of the young adult group. (B) The strain course of the middle-aged group. (C) The strain course of the elderly group.

**Figure 2:**
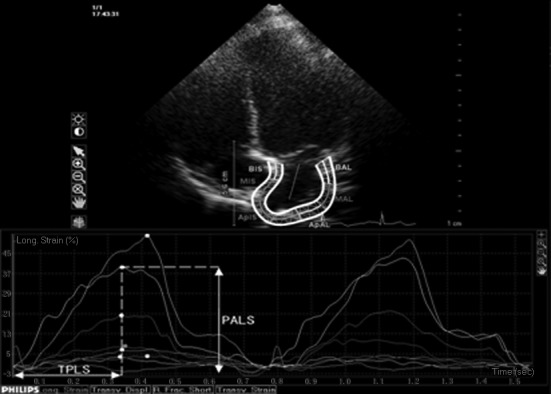
Measurement of peak atrial longitudinal strain (PALS) and time to peak longitudinal strain (TPLS).

The LA strain changes in the inferior segments of each group were higher than the results of the central segments. The changes in the central segments were higher than the results of the superior segments (P<0.05). The strains of the inferior segments of the elderly group decreased as age increased compared with the young adult and middle-aged groups. The central segments of the elderly group were lower than the results of the young adult and middle-aged groups. The young adult and middle-aged groups showed no significant variance. No statistical significance was observed in the superior segment strains of the elderly group as with the other two groups (Table II, Figs. 1 and 2).

As age increased, LA TP of the inferior segment of each wall had a longer duration, which was observed in the elderly group but not with the other two groups (P<0.05). The central segments of the elderly and middle-aged groups were longer than the young adult group (P<0.05). No statistical significance was observed between the middle-aged and elderly groups (P>0.05) as well as in the changes in the superior segment (P>0.05; Table II, Figs. 1 and 2).

## Discussion

LA augments the ventricular reservoir function during atrial contraction, conduit function of early diastole and booster pump function at the end diastole (1). Regardless of physical conditions, LA modulates the LV filling and maintains the normal stroke volume during diastolic dysfunction (5).

The conventional LA function measurement has several approaches (6–8). The most common approaches are tissue Doppler imaging (TDI) and strain rate imaging, which have gained acceptance in the medical field (9,10). These techniques measure the velocity of myocardial motion with accuracy in myocardial diastolic and systolic activities within the cardiac cycle and time and spatial resolution. The surrounding segment and cardiac motions do not affect the results (11). However, these techniques are sensitive to angle-Doppler offset, and require small angle attacks between the acoustic beam and the direction of myocardial motion during measurement. 2DSTI is the latest technology based on the strain and strain rate imaging. The image generated by STI comprises thousands of pixels or acoustic speckles that cover the myocardium uniformly, and form synchronously with myocardial mechanics with no distortion to adjacent images. The STI technique tracks every speckle, calculates the kinetic trajectory in successive frames and provides measurements of velocity, strain and strain rate of the heart tissue. This technique does not use the Doppler principle, therefore, is not sensitive to the angles of attack. 2DSTI provides the longitudinal abnormal motion and the radial and annular activities of the heart, and offers more advantages than the TDI.

LV relaxation and compliance prior to the atrial systole decrease as age increases (12,13), i.e., early peak velocity of the E wave of the mitral valve diastole will drop and the end diastole A wave will increase as measured by a pulse Doppler. In this study, the strain value decreased and TP increased as age increased. These conditions may lead to the possible correlation with the decrease of myocardial elasticity caused by the myocardial stiffness induced by the interstitial fibrosis that resulted from the increase of myocardial interstitial collagen synthesis. In addition, the decrease of LA inferior segment strain and the longer TP were more obvious in the elderly group than in the young adult and middle-aged groups. This condition may be associated with the close location to the mitral annulus, in which the superior segment did not show significant variance for the three groups. This case may be associated with its close location to the PV entrance and far location from the mitral annulus, which was affected less by the LV compliance.

Telagh *et al* discovered that being closer to the mitral annulus leads to a higher peak velocity of the LA (14). The top of the LA is fixed and not involved in the atrial movement and active contraction. In all three groups of this study, the strain of the inferior segment was higher than the central segment, while the central segment was higher than the superior segment. These findings confirm the results of Telagh et al (14). The strain of the inferior segment of the elderly group was markedly lower than the results of the other groups, whereas the central and superior segments did not demonstrate significance. This condition might be associated with the decrease of the LV relaxation and compliance of the elderly volunteers. LA function changes caused by age increase require further study since this study only confirmed that the LA function examination must consider the age factor.

LA function measurements provided novel insight and has prognostic significance for several cardiovascular diseases. STI technique provides an improved sensitivity and conventional measurement of LA function. The proposed technique supported a regular and comprehensive method of examining LA function, assisted early monitoring of sub-clinical diseases and provided useful auxiliary information to clinicians for heart examination.

## Figures and Tables

**Table I:** Comparison of the three age groups.

Variable	Young adult	Middle-aged	Elderly
Age (years), mean ± SD	32.46±8.46	54.01±5.63^a^	70.40±4.76^a,b^
Male, n (%)	30 (57.7)	29 (59.2)	21 (51.2)
Height (cm), mean ± SD	169.13±3.92	168.73±4.19	167.20±3.21
Weight (kg), mean ± SD	60.53±4.94	60.60±3.15	61.46±4.29
Cardiac rate (bpm), mean ± SD	69.33±1.54	71.01±3.02	70.11±2.43
LVEDD (mm), mean ± SD	44.86±2.82	44.93±2.68	44.93±2.78
LVESD (mm), mean ± SD	27.06±1.43	26.33±1.34	26.88±1.74
LVEF (%), mean ± SD	61.01±3.61	60.93±4.39	59.93±4.35
Mitral E/A, mean ± SD	1.56±0.06	1.21±0.11^a^	0.77±0.04^a,b^
E_peak_ deceleration (msec), mean ± SD	171.93±5.39	206.73±8.15^a^	229.26±5.16^a,b^

a P<0.05, compared with the young adult group;

b P<0.05, compared with the middle-aged group. LVEDD, left ventricular end diastole diameter; LVESD, left ventricular end systole diameter; LVEF, left ventricular ejection force; E/A, ratio of mitral valve early diastolic blood flow E peak velocity and end diastolic blood flow A peak velocity.

**Table II:** Comparisons of strain and TP of the three age groups.

Segment	Young adult		Middle-aged		Elderly	
	Strain (%)	Time to peak (msec)	Strain (%)	Time to peak (msec)	Strain (%)	Time to peak (msec)
Inferior	42.2±7.4	389.0±20.0	27.1±4.5^a^	422.6±14.0	25.5±5.5^a,b^	482.8±84.1^a,b^
Central	27.8±7.1	368.5±20.5	25.3±3.8	422.1±37.1^a^	16.3±0.4^a,b^	436.8±31.7^a^
Superior	9.1±3.5	518.0±99.1	8.2±2.9	522.0±56.4	9.6±3.4	526.1±11.8

a P<0.05, compared with the young adult group;

b P<0.05, compared with the middle-aged group. The data are presented as the mean ± SD.
